# Investigations on the Health Status and Infection Risk of Harbour Seals (*Phoca vitulina*) from Waters of the Lower Saxon Wadden Sea, Germany

**DOI:** 10.3390/ani14202920

**Published:** 2024-10-10

**Authors:** Ursula Siebert, Jan Lakemeyer, Martin Runge, Peter Lienau, Silke Braune, Edda Bartelt, Miguel L. Grilo, Ralf Pund

**Affiliations:** 1 Institute for Terrestrial and Aquatic Wildlife Research (ITAW), University of Veterinary Medicine Hannover Foundation (TiHo), 25761 Buesum, Germany; jlow66@yahoo.de (J.L.); mgrilo@ralvt.pt (M.L.G.); 2 Food and Veterinary Institute Braunschweig/Hannover, Lower Saxony State Office for Consumer Protection and Food Safety (LAVES), 30173 Hannover, Germany; martin.runge@laves.niedersachsen.de (M.R.); silke.braune@laves.niedersachsen.de (S.B.); 3 Seehundstation Nationalpark-Haus Norden-Norddeich, 26506 Norden, Germany; pl@seehundstation-norddeich.de; 4 Institute for Fish and Fishery Products Cuxhaven, LAVES, 27472 Cuxhaven, Germany; edda.bartelt@laves.niedersachsen.de (E.B.); poststelle.iff-cux@laves.niedersachsen.de (R.P.)

**Keywords:** harbour seal, pathology, microbiology, virology, parasitology, population health, infectious diseases, zoonosis

## Abstract

**Simple Summary:**

Harbour seals from the Wadden Sea area of Lower Saxony, Germany were investigated for their health status and causes of death for the first time. In newborn seals, weakness and emaciation were the main findings, most likely caused by a separation from the mother. After the weaning period, pulmonary parasitosis and bronchopneumonia were the most frequent pathological findings. These investigations emphasize the importance of a health monitoring programme for this top predator species in the Wadden Sea, as it can provide critical insights into changes in the ecosystem. Monitoring harbour seals, which are sentinel species, will support the implementation of the Trilateral Wadden Sea Agreement and the Marine Framework Directive to protect this important marine environment.

**Abstract:**

Harbour seals (*Phoca vitulina*) are the most common pinniped species in the Wadden Sea of Schleswig-Holstein, Hamburg and Lower Saxony, Germany. Their numbers have recovered after significant depletion due to viral outbreaks and effects of anthropogenic activities like pollution and habitat disturbance. Within the Wadden Sea National Park of Lower Saxony the harbour seal is protected. As a top predator in the Wadden Sea ecosystem, the harbour seal is a sentinel species for the state of the environment. Between 2015 and 2017, a total of 80 stranded dead harbour seals were collected along the coastline of Lower Saxony and submitted for pathological investigations. Of these, 70 seals were born in the same year (0–7 months, age group 1) and eight in the previous year (8–19 months, age group 2), due to high mortality rates in these age groups. However, two perennial animals were also available for examination during this period, one of which was in good nutritional condition. Many of the seals that had been mercy-killed and found dead were in poor nutritional status. Histopathological, microbiological, parasitological and virological examinations were conducted on 69 individuals (86% (69/80)) in a suitable state of preservation. Respiratory tract parasitosis, cachexia, and bronchopneumonia were the most common causes of death or disease. Overall, there was no evidence of a relapse of a viral disease outbreak. Macrowaste, such as plastic waste or fishery-related debris, were not found in any gastrointestinal tract of the animals examined. There was also no evidence of grey seal predation. Weakness and cachexia were prominent causes of disease and death in harbour seals found within a few weeks after birth, but bronchopneumonia and septicaemia also developed in slightly older animals. Frequently found microbial pathogens in seals from Lower Saxony were similar to those found in other studies on seals from the Wadden Sea region in Schleswig-Holstein, for example streptococci and *Escherichia coli*/v. *haemolytica*, *Brucella* spp. and *Erysipelothrix rhusiopathiae*, potentially human pathogenic germs. The results of the examinations of dead harbour seals from Lower Saxony show that pathological investigations on a representative number of animals deliver urgently needed information on the health status of the population. The results represent an important contribution to the state of the top predators of the Wadden Sea as part of the obligations within the Trilateral Wadden Sea Agreement, Oslo and Paris Convention for the Protection of the Marine Environment of the North-East Atlantic (OSPAR) and the Marine Framework Directive. The investigations should be continued as a matter of urgency and the stranding network should be expanded.

## 1. Introduction

The harbour seal (*Phoca vitulina*) is the most common seal species in the Lower Saxon Wadden Sea in northwestern Germany [1]. After hunting was banned in the mid-1970s, the decreased harbour seal population recovered slowly [2]. Also, chemical pollution and habitat disturbance were claimed to have a negative effect in the 1970s and 1980s [3,4]. The seal population was severely devastated and depleted by epidemics in 1988/89 and in 2002 due to phocine distemper virus (PDV), which induced pneumonia and subsequent secondary bacterial infections as the main cause of death [5]. Due to the PDV epidemic in 1988, the total number of harbour seals counted in the Wadden Sea during the moult in August dropped from an estimated 10,000 individuals in 1988 to 4544 in 1989, while the drop in 2002 was from 20,975 to 10,817 [1,5]. In 2014, the harbour seals in the Wadden Sea were struck by an influenza infection (H10N7) for the first time [6,7]. After these outbreaks, the population recovered again and the total number of harbour seals counted in the Wadden Sea during the moult in August 2023 reached 22,621 individuals [1]. Since 1991, harbour seals have been protected under the Agreement on the Conservation of Seals in the Wadden Sea (WSSA) which was concluded under the auspices of the UN Convention on the Conservation of Migratory Species of Wild Animals (Bonn Convention, CMS). The International Union for Conservation of Nature (IUCN) conservation status of the harbour seal in the Baltic Sea protected areas (HELCOM) is highly endangered, while the current status in the North Sea is reported to be endangered (IUCN).

Epidemiological health investigations, especially on the impact of infectious diseases were largely lacking when the first seal die-off started in 1988/89 [5,8]. A stranding network was established for the Wadden Sea coast of Schleswig-Holstein, and systematic pathological investigations were conducted, as increased knowledge on the health status of harbour seals was needed to improve the conservation status [8]. It was demonstrated that harbour seals regularly suffered from parasitic and bacterial infections [6] causing bronchopneumonia, hepatitis, gastroenteritis and septicaemia. Some of the potentially zoonotic bacteria such as *Brucella* spp. or *Erysipelothrix rhusiopathiae* were frequently found in live and dead harbour seals [6].

Human activities, such as shipping [9], offshore-constructions [4], marine litter and microplastic [10,11] as well as chemical pollution [4], may have a negative impact on the growth and health of the Wadden Sea harbour seal populations. Nonetheless, growing grey seal populations may also have a negative influence through grey seal predation and rape [12,13] as well as fatal mating attempts [14] and feeding competition [15].

Contrary to the waters of Schleswig-Holstein, epidemiological information on the health status of harbour seals from Lower Saxony is lacking. Therefore, the Lower Saxony Ministry of Food, Agriculture and Consumer Protection launched a research project allowing researchers to examine seals for their state of health, with special focus on the detection of zoonotic and infectious agents, macrowaste and evidence of grey seal predation. This study aimed to provide results that could be incorporated into the development of infection and hygiene management plans. In addition, the results serve the obligations within the Trilateral Wadden Sea Agreement, Oslo and Paris Convention for the Protection of the Marine Environment of the North-East Atlantic (OSPAR) and the Marine Framework Directive.

## 2. Materials and Methods

**Figure 1:**
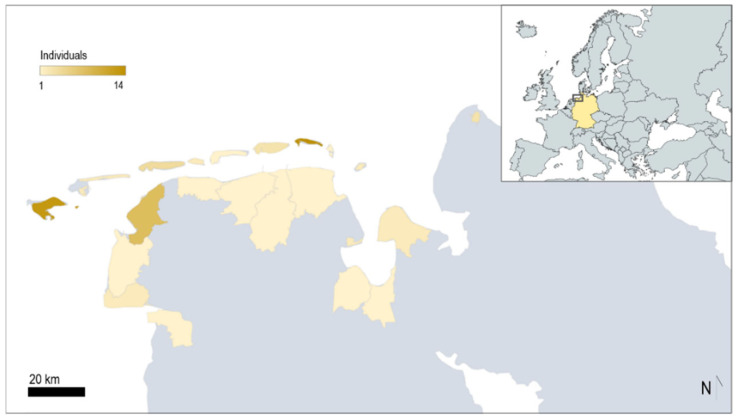
Locations of 80 harbour seals found on the Wadden Sea coast between 2015 and 2017 in the state of Lower Saxony, Germany. Europe map created with mapchart.net (accessed 1 January 2024). Lower Saxony map created with Microsoft Excel 2019 (©Microsoft, Albuquerque, NM, USA).

For this study, the collection of stranded seals on the Wadden Sea coast of Lower Saxony was organised by the Seal Station in Norden-Norddeich (Norden, Germany). This institution is responsible for managing communication with local Wadden Sea Hunting Wardens in charge of handling seals in accordance with Lower Saxony Law (individuals trained by the state of Lower Saxony to monitor seal populations and to perform mercy-killing by shooting when animals present conditions not reversible by the rehabilitation process) and collect dead seals for analysis. The collection period for individuals included in this study (*n* = 80) lasted from January 2015 to May 2017. The project’s timeline included two collection phases: the first began in January 2015 and lasted until June 2015 (*n* = 25) and the second began in July 2016 and lasted until May 2017 (*n* = 55). Locations where seals were found are shown in Figure 1, these ranging from the river Ems in the western coastal part of Lower Saxony to Cuxhaven located in the eastern part of Lower Saxony, including the East Frisian Islands.

Seal carcasses (except for two individuals originating from Döse Beach, Cuxhaven, which were analysed without freezing) were stored at −20 °C in freezers distributed along the coast, at the Seal Station in Norden-Norddeich and at the Institute of Fish and Fishery Products (Cuxhaven, Germany) of the Lower Saxon State Office for Consumer Protection and Food Safety (Wardenburg, Germany). The investigated animals included both seals found dead (36.3%, *n* = 29) and mercy-killed ones (63.7%, *n* = 51). Mercy-killing was either performed by Wadden Sea Hunting Wardens trained by the State of Lower Saxony in the field or by the rehabilitation centre team prior to admission to the rehabilitation centre when the animal did not present a stable condition for a successful rehabilitation and release into the wild. Post-mortem analysis was either conducted at the Institute for Fish and Fishery Products (*n* = 42 seals) or at the Institute for Terrestrial and Aquatic Wildlife Research, University of Veterinary Medicine Hannover (Büsum, Germany; *n* = 38).

**Figure 2:**
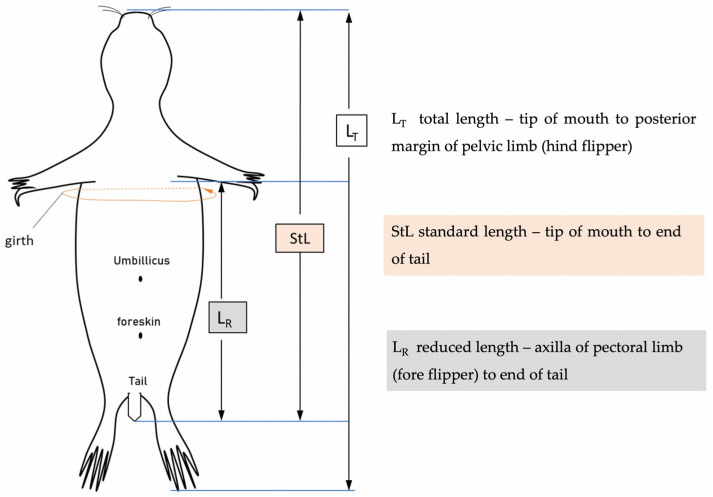
Reference points of the length measurements obtained in the post-mortem analysis of dead seals.

**Figure 3:**
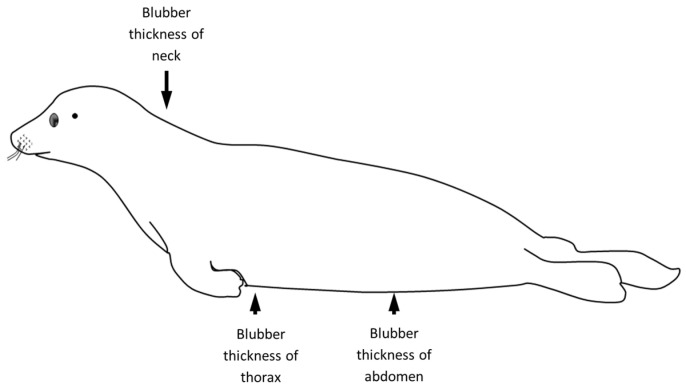
Reference points of the blubber thickness measurements (fatty tissue of the subcutis, blubber) in seals.

Each individual was evaluated in a necropsy examination following guidelines by the European Cetacean Society adapted for necropsies of small cetaceans and seals [8,16]. The status of decomposition of the animals was recorded and scored as follows: 1—fresh; 2—initial autolysis; 3—moderate autolysis; 4—advanced autolysis; 5—severe autolysis [8,16]. The carcasses were weighed and measured (see Figure 2) and the sex of the animal was recorded [8,16]. Age determination was based on the carcass discovery date in relationship to time of year, length and weight [8]. Three age categories were established based on the previous characteristics: (I) animals born in the same year of death (0–7 months); (II) animals born in the year before death (8–19 months); (III) animals older than 19 months (>19 months) [10]. The carcasses were examined for the existence of external lesions, while all available organs were examined macroscopically for specific alterations [8,16]. The nutritional status was based on the individual’s length (total length, standard length and reduced length; Figure 2), body weight, state of the musculature and blubber thickness determined at different locations and divided into good, moderate and emaciated (Figure 3) [8].

For histopathological investigations, samples measuring 1 × 1 × 1 cm^3^ were taken from the lung, trachea, stomach, intestine, heart, liver, pancreas, thyroid and adrenal glands, kidney, urinary bladder, testis, uterus, ovary, spleen, lymph nodes, skeletal muscle, skin and if possible brain and eye and then fixed in 10% buffered formalin and embedded in paraffin wax (Engelbrecht Medizin- und Labortechnik GmbH, Edermünde, Germany). Organ sections were cut (5 µm) and stained with routine haematoxylin and eosin stain (HE). Histopathological analysis was performed at the Institute for Fish and Fishery Products. Parasites detected macroscopically were collected and fixed in 70% ethanol. Prior to identification, parasites were cleared with lactophenol. Identification was performed by light (Leica DM2500 LED, Leica Camera AG, Wetzlar, Germany) and binocular microscopy (Stereo-Zoom-Microscope MSZ5000-T, A. Krüss Optronic GmbH, Hamburg, Germany) [8].

Sampling for bacteriological and mycological analysis was performed either by collection of selected organs or collection of samples with swabs with transport medium (Meus S.r.l., Piove di Sacco, Italy). Sampled organs included brain, heart, intestine (small intestine and colon), kidneys, lungs, liver, reproductive organs, skin and spleen [8,17]. In some cases, swabs were additionally collected from inflamed tissue, for example tracheal mucosa or skin. Organ samples were stored at −20 °C and swabs were stored at 4 °C until further processing to prevent autolysis as well as overgrowth with external bacteria or fungi to maintain the originally sampled microbial flora.

Bacteriological and mycological examinations were carried out at the Veterinary Institute of the Lower Saxony State Office for Consumer Protection and Food Safety (Hannover, Germany). For culture purposes, sampled organs were decontaminated by heating the surface, and fresh cube-shaped sections (approximately 5 mm edge length) were streaked onto agar media. Swabs were plated directly onto the media.

Blood agar containing 5% defibrinated sheep blood (Oxoid Deutschland GmbH, Wesel, Germany) was used as non-selective media, water-blue metachrome-yellow lactose agar (Gassner agar; Oxoid Deutschland GmbH, Wesel, Germany) to detect Enterobacteriaceae. Additionally, selective media were used to isolate *Vibrio* spp. via thiosulphate-citrate-bile salts-sucrose (TCBS) agar (Merck KGaA, Darmstadt, Germany), *Brucella* spp. by use of agar base with *Brucella*-selective supplement (Oxoid Deutschland GmbH, Wesel, Germany), *Erysipelothrix rhusiopathiae* with agar described in [8], *Yersinia* spp. by means of Cefsulodin-Irgasan-Novobiocin (CIN) agar (Oxoid Deutschland GmbH, Wesel, Germany), *Salmonella* spp. through modified semi-solid Rappaport-Vassiliadis (MSRV) agar, brilliant green agar and Xylose-Lysine-Desoxycholate (XLD) agar (Oxoid Deutschland GmbH, Wesel, Germany). *Clostridia* spp. were cultured on Schaedler agar (Oxoid Deutschland GmbH, Wesel, Germany). For selective culturing of fungi Sabouraud agar (Oxoid Deutschland GmbH, Wesel, Germany) was used.

The different culture media were incubated according to the respective methods necessary for culturing each organism. *Brucella* selective agar was incubated in an atmosphere of 10% CO_2_ for at least five days; Sabouraud agar at 28–30 °C for three-14 days (8)S, TCBS agar for two-five days at 25 °C, *Erysipelothrix rhusiopathiae* selective agar medium for 24–48 h at 37 °C, *Yersinia* spp. selective CIN agar for 48 h at 30 °C, MSRV agar for 24 and 48 h at 41 °C, XLD agar for 24 h at 37 °C and Schaedler agar for 24–48 h at 37 °C under anaerobic conditions in a jar using AnaeroGen™ gas sachets (Oxoid Deutschland GmbH, Wesel, Germany). Colonies were subcultured. Pure cultures were identified using standard morphological and biochemical methods as well as matrix-assisted laser desorption ionisation time-of-flight mass spectrometry (MALDI-TOF MS, Bruker Daltonics GmbH & Co. KG, Bremen, Germany). *Salmonella* isolates were identified to the serogroup by agglutination to *Salmonella* antisera (sifin diagnostics GmbH, Berlin, Germany).

Samples for virological analysis were obtained through the collection of tracheal swabs with transport Virocult medium (Sigma-Virocult^®^, Medical Wire & Equipment Co, Corsham, UK). Swabs were stored at 4 °C until investigation. Virological analyses were carried out at the Institute for Virology, University of Veterinary Medicine Hannover, Hannover, Germany. Virological analysis included the determination of influenza viruses, phocine herpesvirus and phocine distemper virus by qPCR [17,18,19,20,21]. RNA extractions for virus were performed using the IndiMag Pathogen Kit w/o plastics (Cat.-No.: SP947257, Leipzig, Germany) with homogenisation of the tissue samples in the BeadMill 24 (Thermo Fisher Scientific Inc., Waltham, MA, USA) using the Lysing Matrix M (MP Biomedicals, LC, Santa Ana, CA, USA) and buffer RA1 + ß-Mercaptoethanol (Macherey-Nagel GmbH & Co. KG, Düren, Germany). All screenings were made by qRT-PCR with the generic morbillivirus primers, as described by [18]. The morbillivirus qRT-PCR was performed with the QuantiTect SYBR Green RT-PCR Kit (Qiagen GmbH, Hilden, Germany). Each reaction consisted of 12.5 μL QuantiTect SYBR Green RT-PCR Mastermix (Qiagen GmbH), 1 μL forward primer (20 pmol) MVP2202, 1 μL reverse primer (20 pmol) MVP2480, 0.25 μL reverse transcriptase, 5.25 μL H2O and 5 μL RNA. Detection of Influenza virus RNA was conducted using the Qiagen QuantiTect Probe RT-PCR kit [19,20]. Details for analyses of phocine herpesvirus are described in [21]. Reactions were performed based on a mixture of 12.5 μL QuantiTect Probe RT-PCR mix, 2.0 μL primer and probe mix (NP-1448-F: 25 μL, NP-1543-R: 25 μL, NP-1473-FAM: 3 μL, H2O: 147 μL), 2 μL GFP mix, 0.25 µL RT-Mix, 3.25 μL H2O, 5 μL sample. For both assays cycling conditions were set at 50 °C at 30 min, 95 °C at 15 min, 40 cycles of 30 s at 95 °C, 30 s at 56 °C and 30 s at 72 °C.

## 3. Results

### 3.1. Origin, State of Preservation, Age and Sex Determination, Morphometric Analyses

In total, 53.7% (*n* = 43/80) of the animals were found stranded in the coastal areas of the mainland of the state of Lower Saxony, while 46.3% (*n* = 37/80) were retrieved in the East Frisian Islands.

**Figure 4:**
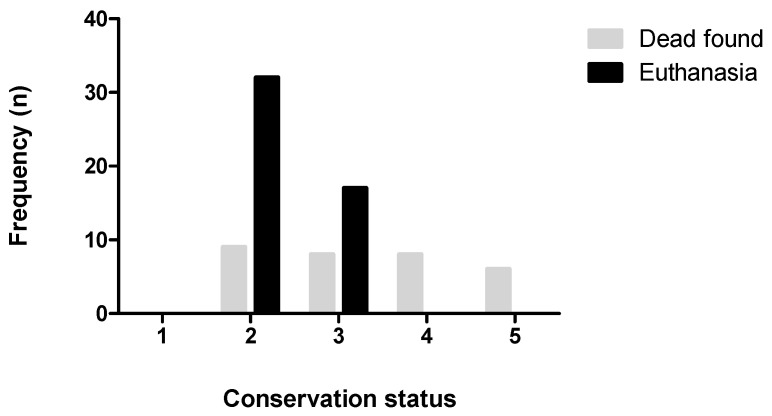
Preservation status of the seals included in this study (*n* = 80): 1—fresh, 2—first signs of autolysis, 3—moderate autolysis, 4—advanced autolysis, 5—macerated or advanced autolysis. Graph produced using GraphPad Prism1 (GraphPad Software, San Diego, CA, USA, version 5.01).

The state of preservation of the animals included in this study is shown in Figure 4. Due to a very advanced decomposition status (state of preservation 5), six (7.5%) of the examined seals were considered unsuitable for histopathological and microbiological analyses. For the eight animals presenting an advanced autolysis (preservation status 4), partial histopathological and microbiological analyses were performed in half of the animals found dead, while in the other half only morphometric data were retrieved. One animal (preservation status 2) was subjected to terrestrial predation and lacked all internal abdominal and most of the thoracic organs and was therefore excluded from the pathological analysis. In total, 69 animals (86%) were evaluated.

Sex and age determination were performed for all individuals, while morphometric data were retrieved when possible (Table 1). From the studied sample, 87.5% (*n* = 70/80) were individuals born in the same year they died, 10% (*n* = 8/80) had been born in the previous year, and 2.5% (*n* = 2/80) were older than 19 months. Most individuals were females (58.75%, *n* = 47/80).

### 3.2. Assessment of Health Status

#### 3.2.1. Nutritional Status

From all 80 individuals, the majority of examined seals presented an emaciated status (67.5%, *n* = 54/80). These animals presented cachexia and severe muscle atrophy. Most of the individuals were younger than six months. Overall, 25% (*n* = 20/80) of the individuals presented a good nutritional status.

#### 3.2.2. Pathological Findings

Pathological findings collected during necropsies of the 69 examined seals are summarised in Table 2.

##### Respiratory System

**Figure 5:**
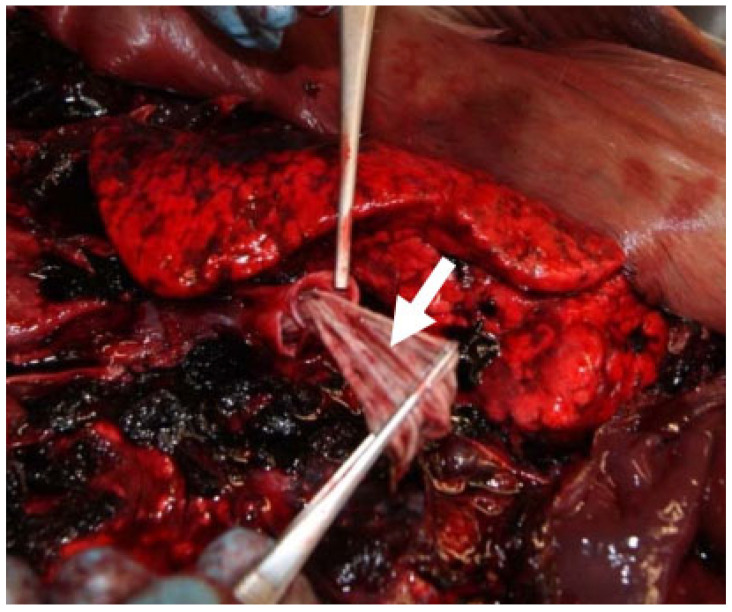
Severe lungworm infection with complete obstruction of the right main bronchus by pulmonary nematodes (arrow).

**Figure 6:**
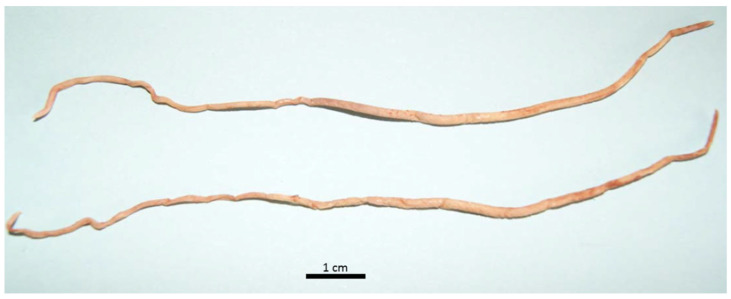
Roundworms (*Otostrongylus circumlitus*) isolated from the trachea and fixed in 70% ethanol.

In 29 examined seals (42%), a nematode infection was observed in the bronchial tree (Figure 5). The majority of respiratory parasitic infections were recorded in the younger individuals (age class I, *n* = 22), while no cases were recorded in the older animals (age class III). Respiratory parasitosis ranged in terms of severity presentation (mild to severe). Two animals in the age classes I and II presented minor parasitic infections, seven had moderate parasitic infections, and the majority (*n* = 20) displayed severe parasitic infections in the respiratory system. This finding was particularly evident in animals of age class I (n = 16) which simultaneously presented good nutritional status. Parasites retrieved from the trachea (Figure 5), large main bronchi and smaller pulmonary bronchi were identified as roundworms *Otostrongylus circumlitus*. The largest nematodes (Figure 6) were up to 14.5 cm long (average) and had a diameter of 2 mm (unfixed). Often, lungworms were also isolated from the larynx and stomach.

**Figure 7:**
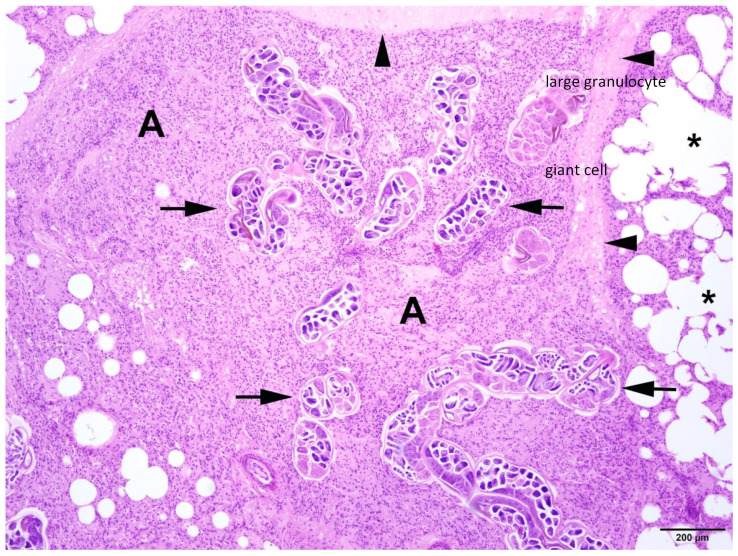
Harbour seal, lung: severe pulmonary endoparasitosis with nematodes in the parenchyma (arrows) associated with lobular atelectasis (A). Additionally, there is a moderate interstitial oedema (arrowheads) and an alveolar emphysema in the adjacent pulmonary tissue (asterisks). HE (4×).

**Figure 8:**
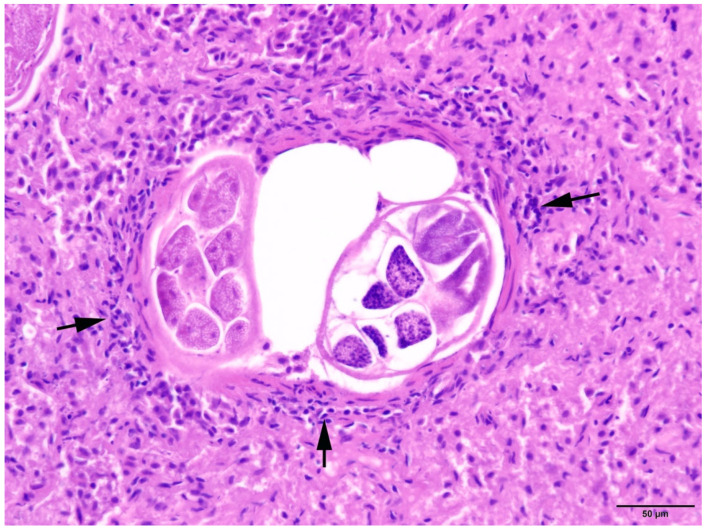
Harbour seal, lung: Bronchiolus with intraluminal nematodes and mild chronic peribronchitis (arrows). HE (20×).

In all animals with respiratory parasitic infections, bronchopneumonia or pneumonia was detected. Several types of inflammation could be diagnosed, including catarrhal-purulent or -haemorrhagic, granulomatous-necrotising, purulent-necrotising, and alveolar-interstitial. In most cases, pathological changes included several types of inflammatory lesions within the same animals (Figure 7 and Figure 8).

The presence of red-grey-brown marbled lungs were associated with respiratory parasitic infections and/or bronchopneumonia, while tracheitis was recorded in eight individuals. Bilateral red-grey-brown marbled lungs were remarkably frequent, often resulting from agonal blood aspiration secondary to lesions existent in the lung tissue. Most cases were recorded in age class I animals (*n* = 21) and to a lesser extent in animals in age class II (*n* = 8), which constitute the individuals presenting the more severe respiratory parasitic infections.

**Figure 9:**
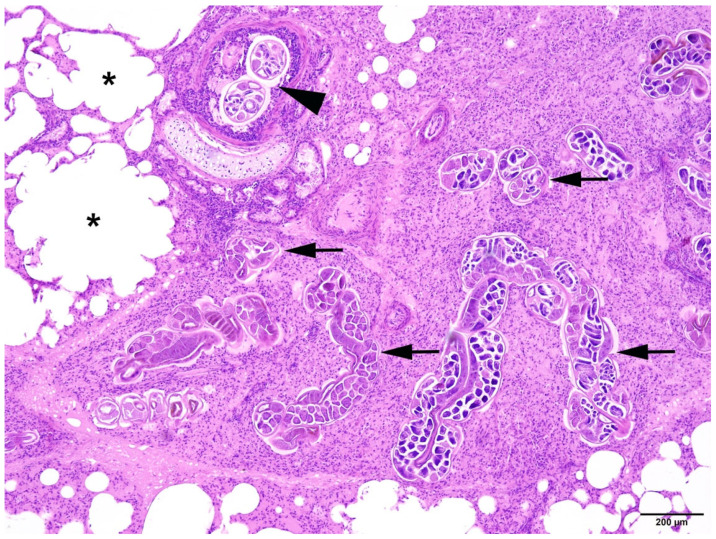
Harbour seal, lung: severe pulmonary endoparasitosis with nematodes in a bronchus (arrowhead) and in the parenchyma (arrows) with associated atelectasis. Adjacent pulmonary tissue displays alveolar emphysema (asterisks). HE (4×).

Different severity grades of alveolar/interstitial pulmonary emphysema (pathological accumulation of gas in the alveoli and/or cell spaces) could be detected in 44 animals often associated with parasitic infection and bronchopneumonia (Figure 9). Pulmonary atelectasis (collapse of alveoli) was found in 21 individuals. In 46 animals, alveolar/interstitial pulmonary oedema (pathological fluid accumulation in the alveoli and/or cell spaces) was recorded, with alveolar oedema present in 37 animals.

##### Cardiovascular System

Parasitic infections by *Otostrongylus circumlitus* were detected in the heart of eight seals (12%). The nematodes were mostly located in the right side of the heart (atrium and ventricle). Five animals in age class I (representing around 7% of the animals in this age class) presented a persistent ductus arteriosus. After incision, it was observed that the blood vessel was obstructed by a white-greyish mass. Differentiation between a physiological obstruction or a thrombus (i.e., white thrombus, separation thrombus) could not be concluded. One animal showed signs of periarteritis.

##### Gastrointestinal System

**Figure 10:**
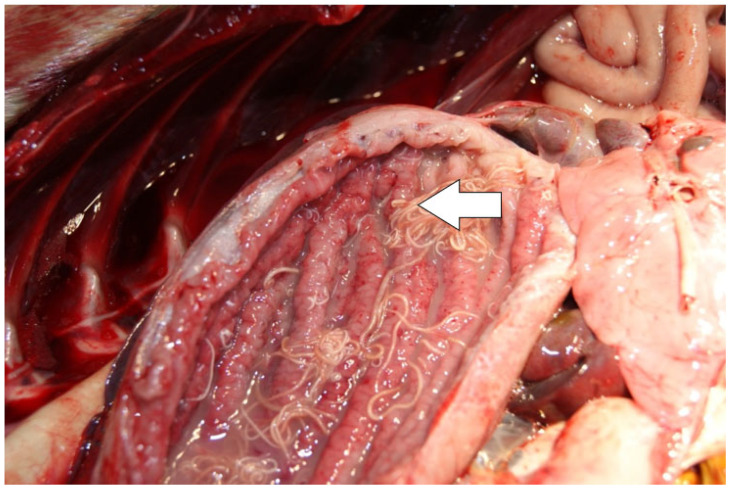
Severe gastric parasitic infection. Several roundworms (arrow) were found in the lumen of the stomach. The gastric mucosa is hypertrophied, forming broad increased folds and shows signs of petechiation (pin-point sized haemorrhages).

In several animals, parasitic nematodes were retrieved from the gastrointestinal system (stomach: *n* = 4 and intestines: *n* = 7). However, it was not completely clear in some cases whether these findings corresponded to gastrointestinal parasites (*Pseudoterranova decipiens* and/or *Contracaecum osculatum*) or to pulmonary parasites (*Otostrongylus circumlitus*) that were coughed up and swallowed by the animal while it was alive. Ultimately, the simultaneous presence of morphologically similar parasites in the respiratory system and in the gastrointestinal system, without observed parasite attachment in the gastrointestinal mucosa and associated lesion patterns, was considered evidence for this phenomenon. Figure 10 shows a gastric parasitic infection. In eight animals (12%), gastritis was present. Half of these animals presented a concomitant gastric parasitic infection.

**Figure 11:**
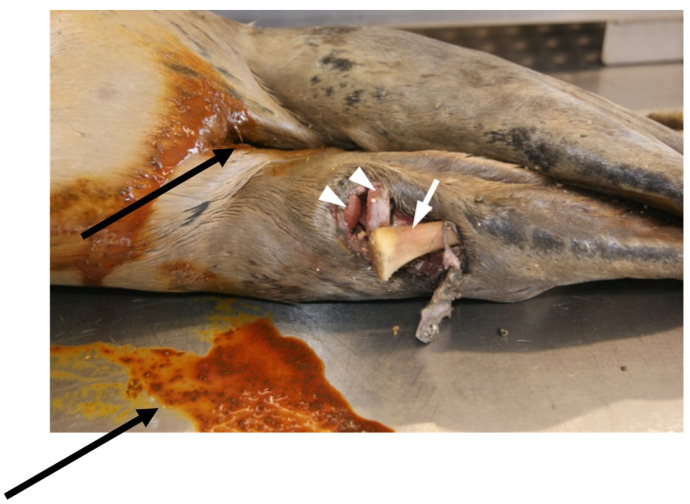
Seal, ventral view. Perianal area contaminated with diarrhoea (black arrows). Posterior right flipper showing an open fracture involving the tarsal and metatarsal bones, with partial shattering, bone dislocation and dislocated fracture ends. In the fracture area, there is severe skin and subcutaneous tissue loss (10 × 8 cm). One of the metatarsal bones (white arrowheads) protrudes from the open wound, while parts of the tarsal bones (white arrow) can also be observed.

In 15 seals (22%), enteritis was evident, with the intestines presenting inflammation of the mucosa. In 12 of these seals, the affected animals also presented diarrhoea (unformed faeces) in the rectal area, with extensive contamination of the perianal area (fur and skin) (Figure 11). Regarding the underlying causes for enteritis, inflammatory and malabsorption statuses (the latter secondary to prolonged starvation) were considered.

Regarding findings in the liver, hepatic congestion due to passive congestive hyperaemia was observed in 17 (25%) animals. Most individuals where this alteration was detected belonged to age group I. Three animals (4%) presented hepatic steatosis, and hepatic haemorrhage was detected in two individuals (3%). Hepatitis was diagnosed in one animal (1%), and jaundice in another animal (1%).

##### Kidney, Reproductive Organs, Navel

**Figure 12:**
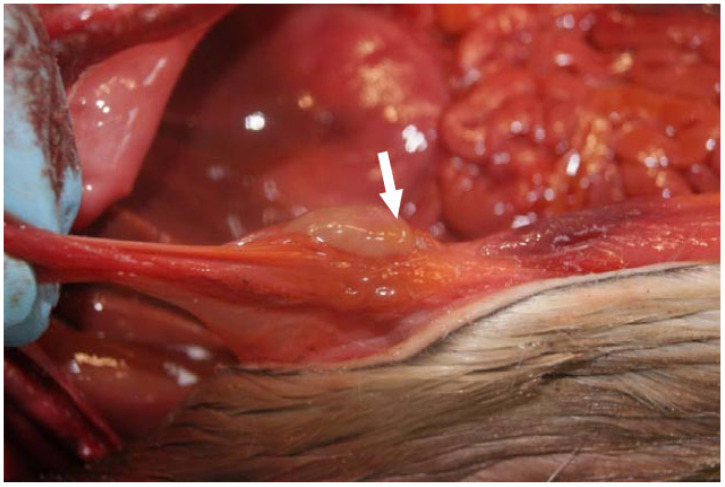
Seal, suppurative inflammation of the navel; when cut, a yellowish-grey and slightly viscous mass protruded (umbilical abscess, arrow).

In 10 animals from age group I, partial or complete patency of the navel was found, with three of these animals showing an infection of the navel. In another case, a firm, movable, 0.5 cm increase in circumference was found in the navel area. During microscopic examination, scar tissue was found, presumably because of a past infection (Figure 12). In three female harbour seals, inflammation of the uterus and vagina as well as bleeding in the vulva/vagina were encountered. In one of these three animals, the inflammation of the uterus encompassed the endo- and myometrium. Inflammation of the glans penis was found in three male animals. Urinary bladder inflammation was diagnosed in two animals. Another three seals had grit in the bladder.

##### Eyes, Ears, Central Nervous System (CNS)

One animal was missing both eyes due to bird scavenging damage, one seal displayed catarrhal conjunctivitis on both eyes and another animal had bilateral corneal ulcers.

##### Skeletal Muscles

All cachectic seals (*n* = 44) showed reduced blubber as well as muscle atrophy, of which 41 (93.2%) belonged to age group I.

##### Joints and Bones

Diffuse purulent arthritis was located in one animal. Another animal had an open comminuted fracture of the right rear flipper in the area of the tarsal and metatarsal bones with luxation of the metatarsalia—tarsalia (Figure 10) associated with 5–10 cm long, 4–8 cm wide and 0.5–1 cm deep skin wounds of reddish-brownish colour in the fracture area. In the case of another seal, the perforations of the os occipitale and os interparietale of the skull were of unknown origin. Furthermore, one animal developed a joint abscess of the tarsal joint.

##### Skin and Hypodermis

**Figure 13:**
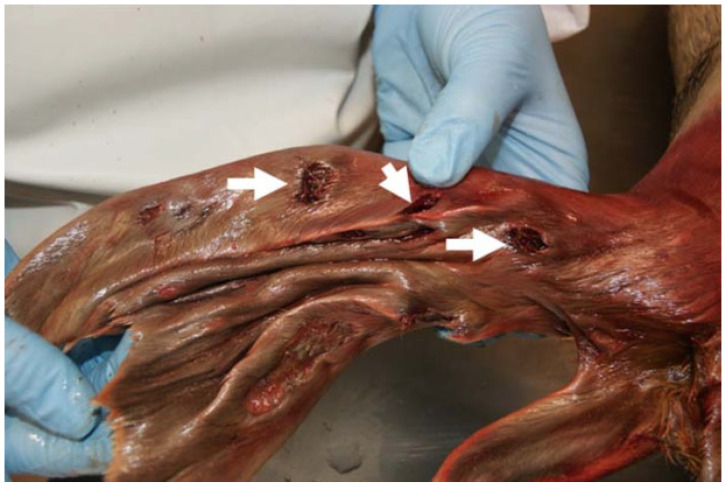
One seal showed superficial to 0.5–2 cm lesions on different parts of the skin (polytrauma); in the plantar metatarsal region of the right posterior flipper, there are clearly visible, fresh, up to 10 euro cent-sized, round-ovoid, bleeding, 0.2–0.5 cm deep skin wounds (arrows).

Four of the seals examined showed wounds on the skin of the limbs and abdominal skin, varying in size between 0.5–8 cm length, 0.5–2 cm width and 0.2–0.7 cm depth (Figure 13). Subcutaneous haematomas on the abdominal wall and head were found in six harbour seals.

### 3.3. Bacteriological and Mycological Results

Various tissues (brain, eye, skin/subcutaneous tissue, sexual organs, intestines, bladder, liver/bile ducts, lungs, heart, navel, kidneys, lymph nodes) and various swab samples (including lungs, musculoskeletal system, skin) were sampled from the necropsied animals. In the 73 seals examined, this corresponded to 730 microbiological samples.

Among the potentially pathogenic bacteria, α- and ß-haemolytic were the most common streptococci, including *Streptococcus* (*S*.) *phocae*. Furthermore, *Escherichia coli* and *Escherichia coli* variatio (var.) *haemolytica* were diagnosed frequently (Table 3). The bacteria detected were associated with various diseases, especially bronchopneumonia and septicaemia. *Arcanobacterium phocae* could also be detected in the investigated harbour seals. *Brucella* (*B*.) *pinnipedialis* was isolated from the organs of three harbour seals. Human pathogenic bacteria such as *Erysipelothrix rhusiopathiae* and *Salmonella enterica* subsp. *enterica* ser. *typhimurium* were isolated from the organs of two seals each.

Other bacteria such as *Acinetobacter* sp., *Clostridium perfringens*, *Moraxella* sp., *Pantoea* sp., *Proteus* sp., *Pseudomonas* sp. and *Serratia* sp. were also detectable in various organs. In addition to bacteria, yeast (*Candida* sp.) and mold (*Aspergillus fumigatus*) were diagnosed. All findings are listed in Table 3.

### 3.4. Virological Results

No signs of viral infection were observed during necropsies and histopathological examinations. A total of 73 seals were tested for seal distemper, seal herpes virus and influenza virus by means of PCR. PCRs provided negative diagnostics in all animals.

### 3.5. Parasitological Investigations

Of the 69 seals examined for parasites, 29 displayed parasitic infections in the respiratory system. The animals affected by lungworms were especially the yearlings (*n* = 22) with a body mass between 6 and 19 kg (for further details, see Table 4). The macroscopic and microscopic examinations showed an infection of the respiratory tract with *Otostrongylus circumlitus*. The infection was regularly concomitant with pneumonia or bronchopneumonia. Heavy parasitic infection of the lung also resulted in *Otostrongylus circumlitus* found in the heart, stomach and intestine through coughing and swallowing.

### 3.6. Causes of Disease and Death

The majority of the seals shot and found dead had a bad nutritional status. This could have been caused by infectious diseases, general debility in young animals or loss of the mother. Signs of septicaemia, such as generalised intravascular thrombi present as a result of DIC (disseminated intravascular coagulopathy), petechial haemorrhages in serous membranes as well as generalised or multiple organ infarctions were not observed in any of the necropsied animals. The most common causes of disease and death in the harbour seals of the Lower Saxon dead animal monitoring were parasitosis of the respiratory tract, followed by cachexia and bronchopneumonia.

## 4. Discussion

The health assessment of harbour seals was mainly based on dead animals, with examinations of yearlings (*n* = 70) and animals born in the year before their death (*n* = 8). This is due to the high morbidity and mortality rates in these age classes, affecting many harbour seal populations [1,5]. However, during this period there were also two perennial animals for examination, one of which was in good preservation status.

Most of the mercy-killed seals and those found dead displayed poor nutritional status and health. The main causes were infectious diseases and weakness during life in young animals, most likely due to maternal separation. Separation during the lactation period can be caused by human disturbances or the illness or death of the mother. In order to minimize human–seal interaction, the National Park authorities of the Wadden Sea, the seal stations, the Common Wadden Sea Secretariat and exhibition centres regularly provide information about the sensitivity of the newborn animals [1].

Disease and death in the examined harbour seals were especially induced by parasitosis of the respiratory tract, cachexia and bronchopneumonia. Those findings are in accordance with harbour seals examined in other regions in the North and Baltic Seas as well as North America [6]. Parasitosis of the respiratory tract and bronchopneumonia are also the most common pathological findings in harbour porpoises in the North Sea [22,23,24].

Overall, there was no evidence of a renewed, epidemic virus-related seal die-off neither in Lower Saxony nor in Schleswig-Holstein. However, combined health examinations of dead and alive seals in Schleswig-Holstein have shown that certain parameters, such as blood values [25], antibody and antigen detection for distemper [17] and influenza virus [9] should also be conducted in living animals in Lower Saxony in order to ensure a likewise assessment of older seals. Given the ongoing H1N5 influenza infections in wild bird populations, it can be expected that more positive cases will be found in marine mammals in the North Sea.

No macrowaste, such as plastic debris or fishing nets, was found in the gastrointestinal tract or on the fur, as described for animals from Schleswig-Holstein and Mecklenburg-Western Pomerania [10]. In the future, microplastic analyses should be introduced for animals from Lower Saxony, as previous analyses in different regions showed contamination by microplastic, which is of potential concern [11,26,27].

Furthermore, there were no characteristic indications of injuries from predators, as frequently found in Schleswig-Holstein [12,13] or harbour porpoises from Dutch and Belgian waters [28,29]. The lesions observed in the harbour seals from Lower Saxony are most likely due to bites from other seals or possibly dogs or blunt trauma.

When comparing the examination results with those of harbour seals from Schleswig-Holstein, it becomes clear that the examination material was also dominated by yearlings and the previous year’s animals [8]. In the animals found around the period of birth, general debility and cachexia were also major causes of disease and death. Bronchopneumonia and septicaemia affected slightly older animals as well. Frequently found microbial pathogens in Schleswig-Holstein were also streptococci and *Escherichia coli* var. *haemolytica* [6].

This study detected *B*. *pinnipedialis* in the gonads of Lower Saxon harbour seals for the first time. The importance of *B*. *pinnipedialis* as a causative agent of marine mammal diseases and the risk potential as zoonotic pathogen are discussed in further research studies [8]. With *Brucella* sp. [6,30,31] and *Erysipelothrix rhusiopathiae* [32,33], potentially human pathogenic germs were found in both the states of Lower Saxony and Schleswig-Holstein, which always admonish to adhere to a careful handling of dead and alive seals.

*Streptococcus phocae* was also isolated during disease outbreaks on a salmon farm in 2005 [34]. This pathogen was first identified in 1997 in harbour seals in Scotland and later also in other marine species isolated from skin lesions such as wounds and abscesses [35]. In marine mammals, the pathogen was proven for the first time in 1994 [8,36]. Overall, streptococci are important pathogenic bacteria in marine mammals, also including zoonotic species such as *Streptococcus halichoeri* and *S*. *iniae* [36], warranting close monitoring.

A handout was compiled for the training of the Wadden Sea Game Wardens, which was used as part of the training courses at the seal station Norddeich. The information about the project should also be included in future exhibitions in the seal station and the national park buildings. The results of this study, which involved post-mortem examinations of a representative sample of dead harbour seals from Lower Saxony, contribute valuable insights into the health status of these top predators in the Wadden Sea. These findings will support efforts under the Trilateral Wadden Sea Agreement, OSPAR, and the Marine Framework Directive. These investigations should be continued, and the stranding network expanded as a matter of urgency.

## 5. Conclusions

This study underscores the importance of systematic pathological investigations, as basic knowledge of the health status and causes of death are needed to ensure the discovery of known infectious agents, including zoonotic pathogens. The causes of die-offs are identified more quickly when the health status of animals in specific areas is known. In addition, knowledge of infectious agents informs necessary adaptations to hygiene protocols when handling seals. Pathological investigations help to understand changes in the health of the Wadden Sea harbour seal population living in an environment affected by anthropogenic impacts like shipping, offshore-construction, ammunition, chemical pollution and marine litter. These results should encourage stakeholders to conduct annual health monitoring, as long-term studies are needed to understand the impacts of climate change, pollution, human activity, and habitat disruption, and to ensure adapted future management and protection strategies.

## Figures and Tables

**Table 1:** Sex, morphometry and nutritional status data of examined seals in relation to the age class. ^1^ Mean and standard deviation, ^2^ Total count, ^3^ Coefficient of variation, ^4^ Minimum and maximum values.

		Age Classes			
		I 0–6 Months	II 7–19 Months	III >19 Months	Total
**Sex**	Male	28 (35%)	5 (6.25%)	0 (0%)	33 (41.25%)
	Female	42 (52.5%)	3 (3.75%)	2 (2.5%)	47 (58.75%)
	Total	70 (87.5%)	8 (10%)	2 (2.5%)	80 (100%)
**Morphometry**	Body weight BW (kg)	10.06 ± 4.90 ^1^ (70) ^2^	18.21 ± 2.92 (8)	62.80 ± 3.69 (2)	80 (100%)
		48.7% ^3^	16.0%	6.3%	
		5.20–18.80 ^4^	12.4–21.9	60.0–65.5	
	Total length L_T_ (cm)	89.28 ± 8.84 (67)	105.16 ± 6.42 (8)	158.00 ± 1.41 (2)	77 (96.25%)
		9.1%	6.1%	0.9%	
		72–115	94.3–112.0	157.0–159.0	
	Standard length L_St_ (cm)	77.66 ± 9.60 (67)	94.50 ± 6.70 (8)	137.0 (1)	76 (95%)
		12.4%	7.1%	-	
		45–100	80.5–100.0	-	
	Reduced length L_red_ (cm)	50.32 ± 5.98 (69)	60.69 ± 4.64 (8)	86.5 (1)	78 (97.5%)
		11.9%	7.6%	-	
		42.00–70.00	50.5–64.5	-	
	Girth axilla A (cm)	48.98 ± 9.51 (67)	67.25 ± 5.98 (8)	99.0 (1)	76 (95%)
		19.4%	8.9%	-	
		38.0–66.0	56.0–77.0	-	
	Blubber thickness thorax (mm)	7.7 ± 3.8 (67)	12.13 ± 5.00 (8)	40.0 (1)	76 (95%)
		49.5%	41.2	-	
		0.0–15	6.0–22.0	-	
	Blubber thickness neck (mm)	7.9 ± 4.1 (66)	12.38 ± 3.02 (8)	33.0 (1)	75 (93.75%)
		51.5%	24.4%	-	
		0.0–18.0	8.0–17.0	-	
	Blubber thickness abdomen (mm)	6.4 ± 3.4 mm (34)	11.40 ± 5.99 (5)	-	39 (48.75%)
		52.6%	52.5%	-	
		1.0–13.0	10.0–13.0	-	
**Nutritional status**	Good	14 (17.5%)	5 (6.25%)	1 (1.25%)	20 (25%)
	Moderate	6 (7.5%)	0 (0%)	0 (0%)	6 (7.5%)
	Emaciated	50 (62.5%)	3 (3.75%)	1 (1.25%)	54 (67.5%)
	Total	70 (87.5%)	8 (10%)	2 (2.5%)	80 (100%)

**Table 2:** Pathological findings in examined harbour seals (*n* = 69).

System/Region	Organ	Pathological Findings	Frequency
Respiratory system	Lungs and Bronchi	Pulmonary oedema	46 (67%)
		Pulmonary emphysema	44 (64%)
		Pulmonary parasitic infection	29 (42%)
		Bronchopneumonia/pneumonia	29 (42%)
		Atelectasis	21 (30%)
		Pulmonary haemorrhage	8 (12%)
		Congestion/hyperaemia	3 (4%)
	Larynx	Oedema	1 (1%)
	Trachea	Tracheitis	8 (12%)
		Oedema	1 (1%)
Thoracic cavity	Mediastinum	Mediastinal haemorrhage	9 (13%)
		Mediastinal emphysema	9 (13%)
Cardiovascular system	Blood vessels	Patent ductus arteriosus	5 (7%)
		Periarteritis	1 (1%)
	Heart	Auricular and ventricular parasitic infection	8 (12%)
		Right-sided cardiac failure	2 (3%)
		Perforation of the heart (secondary to gunshot)	1 (1%)
Gastrointestinal system	Teeth	Teeth deformation	1 (1%)
	Stomach	Gastritis	8 (12%)
		Gastric parasitic infection	4 (6%)
		Gastric haemorrhage	3 (4%)
	Intestines	Intestinal parasitic infection	7 (10%)
		Enteritis	15 (22%)
		Diarrhoea	12 (17%)
	Liver and Bile ducts	Hepatic congestion	17 (25%)
		Hepatic steatosis	3 (4%)
		Hepatic haemorrhage	2 (3%)
		Hepatitis	1 (1%)
Reproductive and Urinary system	Vagina and Uterus	Vaginitis/vulvitis	3 (4%)
		Endometritis/myometritis	1 (1%)
	Penis	Balanoposthitis	3 (4%)
	Umbilicus	Incomplete closure	10 (14%)
		Omphalitis	7 (10%)
	Kidneys	Renal congestion	3 (4%)
		Subcapsular renal haematoma	2 (3%)
		Renal cysts	1 (1%)
	Bladder	Urine sediment	3 (4%)
		Cystitis	2 (3%)
Locomotor system	Musculature	Muscular atrophy	44 (64%)
		Subcutaneous haematoma	6 (9%)
	Bones and articulations	Arthritis/polyarthritis	1 (1%)
		Open comminuted fracture with dislocation	1 (1%)
Skin and subcutis	Skin	Wounds of the skin and subcutis	1 (1%)
Haematopoietic and endocrine system	Spleen	Hyperplasia of the white pulp	4 (6%)

**Table 3:** Findings of the microbiological examinations in different organs in 73 harbour seals from Lower Saxony, Germany. Number indicates the bacterial or fungal isolates.

	Organ											
Bacteria and Fungi	Intestine	Genitalia	Skin	Heart	Liver	Lung	Spleen	Organ Pool	Swabs	CNS	Total Results	Number of Harbour Seals
Number of investigated harbour seals												73
Bacteriological und mycological differentiations:												
*Acinetobacter pittii*						1				1	2	1
*Acinetobacter lwoffii*				1	1	1					3	1
*Acinetobacter johnsonii*				1		1					2	1
*Acinetobacter* sp.				1							1	1
*Aerococcus viridans*				3	3	4					10	4
*Arcanobacterium phocae*				1		2					3	2
*Arcanobacterium phocisimile*				1		2					3	3
*Aspergillus fumigatus*				1							1	1
Unknown bacterium	1			2		1					4	4
*Brevundimonas diminuta*				1							1	1
*Brucella pinnipedialis*		2			2						4	3
*Burkholderia cepacia*				1							1	1
*Candida zeylanoides*			1								1	1
*Carnobacterium maltaromaticum*				2	2	2					6	2
*Clostridium bifermentans*	5										5	5
*Clostridium perfringens*	46										46	46
—of these *Clostridium perfringens* type A	38										38	38
—of these *Clostridium perfringens* type D	1										1	1
*Clostridium sordellii*	9										9	9
*Clostridium* sp.	1										1	1
*Clostridium tertium*								1			1	1
Coliform bacteria				3	3	3					9	3
*Corynebacterium jeikeium*				1		1					2	1
*Corynebacterium pseudodiphteriticum*				1	1						2	1
*Cronobacter malonaticus*				1	1	1					3	1
*Enterobacter cloacae*				1	1						2	1
*Enterobacter* sp.				1	1	1					3	1
*Enterococcus avium*				3	1	2					6	3
*Enterococcus durans*					1						1	1
*Enterococcus faecalis*	2			7	5	6					20	11
*Enterococcus faecium*				1	1	1					3	2
*Erwinia herbicola*					1						1	1
*Erysipelothrix rhusiopathiae*								2			2	2
*Escherichia coli*	1			5	4	6					16	6
*Escherichia coli* (haemolytic)				4	3	3					10	5
*Ewingella americana*						1					1	1
*Gardnerella vaginalis*				3		2					5	3
*Gemella haemolysans*				1	1	2					4	2
*Gemella morbillorum*				1		2					3	2
*Hafnia alvei*				1	1	1					3	1
Yeasts			1								1	1
*Moraxella* sp.				3	3	3					9	3
*Morganella morganii*					1						1	1
Indistinguishable germs				2	3	2					7	5
Indistinguishable germs (fungus)			1								1	1
*Oceanobacillus oncorhynchi*					1						1	1
*Pantoea* sp.				1							1	1
*Pasteurella* sp.				1		1					2	1
*Penicillium* sp.			1								1	1
*Pseudomonas aeruginosa*						1					1	1
*Pseudomonas fluorescens*				6	6	6					18	8
*Pseudomonas lundensis*				2	2	2					6	3
*Pseudomonas proteolytica*					1						1	1
*Pseudomonas putida*				3	3	3					9	4
*Pseudomonas* sp.				1	1	1					3	1
*Psychrobacter phenylpyruvicus*				3	4	2					9	4
*Rahnella aquatilis*				1	1		1	1			4	3
*Raoultella ornithinolytica*	1										1	1
*Salmonella typhimurium*	2			1	1	1		1			6	2
*Serratia grimesii*				2							2	2
*Serratia liquefaciens*				4	3	4					11	4
*Serratia marcescens*	1			1	1	1					4	2
*Serratia plymuthica*				1		1					2	1
*Serratia* sp.				1	1	1					3	1
*Serratia ureilytica*				1	1	1					3	1
*Staphylococcus* sp.				2	3	3					8	4
*Streptococcus equi* spp. *equi*									1		1	1
*Streptococcus gallolyticus*						1					1	1
*Streptococcus phocae*				14	7	19					40	19
*Streptococcus sanguinis*				1	1	1					3	1
*Streptococcus* sp.						1					1	1
*Torulaspora delbrueckii*			1								1	1
*Vagococcus fluvialis*				1	1	1					3	1
*Vibrio alginolyticus*	1							1			2	1
Anaerobic bacteria negative	18										18	18
Brucella negative		64			66			70		69	269	71
Francisella negative					71		71				142	71
*Erysipelothrix rhusiopathiae* negative								69			69	69
Fish pathogenic pathogens (Flavobacteria, non-motile *Aeromonas*)				71	70	71					212	71
*Salmonella* negative	66							69			135	69
*Vibrio* negative	68							70			138	70
*Yersinia* negative	70				71		71				212	71
Non-specific bacteria									1		1	1
Bacterial investigation negative				26	26	21					73	29
Mycological investigation negative			8								8	8
Total results	400	66	13	198	400	245	143	284	2	70	1821	46

**Table 4:** Number, sex ratio, body weight and standard lengths of parasites in the respiratory system of investigated harbour seals (♂ = male, ♀ = female).

Age	Body Weight [kg]	Total Length [cm]	Standard Length [cm]	Number N	Sex Ratio
age class 1	13.1 ± 2.5	96.6 ± 8.1	85.1 ± 8.0	22	9 ♂; 13 ♀
age class 2	17.7 ± 2.7	104.8 ± 6.8	93.7 ± 6.8	7	4 ♂; 3 ♀

## Data Availability

The data supporting the conclusions of this article are available upon request to the authors.
